# Prognostic Role of CA19-9 in Patients Undergoing Hepatectomy for Colorectal Liver Metastases

**DOI:** 10.3390/cancers18101624

**Published:** 2026-05-17

**Authors:** Toshiro Masuda, Toru Beppu, Tatsunori Miyata, Hirohisa Okabe, Katsunori Imai, Katsunori Sakamoto, Yuji Miyamoto, Hiromitsu Hayashi

**Affiliations:** 1 Department of Surgery, Yamaga City Medical Center, Yamaga 861-0593, Japan; masuda1046@yahoo.co.jp; 2 Department of Gastroenterological Surgery, Graduate School of Life Sciences, Kumamoto University, Kumamoto 860-8556, Japan; tmiyata4@kumamoto-u.ac.jp (T.M.); hirohisaokabe1251@yahoo.co.jp (H.O.); katunori-imai@saiseikaikumamoto.jp (K.I.); miyamotoyuji@gmail.com (Y.M.); hhayasi@kumamoto-u.ac.jp (H.H.); 3 Division of Hepato-Biliary-Pancreatic Surgery and Transplantation, Department of Surgery, Graduate School of Medicine, Kyoto University, Kyoto 606-8507, Japan; sakamo87@kuhp.kyoto-u.ac.jp

**Keywords:** colorectal liver metastases, carbohydrate antigen 19-9, carcinoembryonic antigen, prognostic biomarkers, hepatectomy, recurrence-free survival, recurrence-risk stratification, tumor markers, recurrence-risk stratification

## Abstract

Carbohydrate antigen 19-9 (CA19-9) is widely used as a tumor marker in gastrointestinal malignancies; however, its clinical significance in patients undergoing resection for colorectal liver metastases (CRLM) remains unclear. This review summarizes current evidence regarding the prognostic value of CA19-9 in CRLM, with particular emphasis on its role compared with carcinoembryonic antigen (CEA) and its integration into modern prognostic models. Across multiple cohort studies, elevated preoperative CA19-9 levels have consistently been associated with worse recurrence-free and overall survival after hepatectomy. In several multivariable analyses, CA19-9 emerged as a significant prognostic factor whereas CEA did not. CA19-9 has also been incorporated into several prognostic scoring systems and nomograms, including the JSHBPS nomogram (Beppu score) and the Imai nomogram, highlighting its value as a surrogate marker of potentially unfavorable tumor biology. Reported cutoff values vary widely across studies, ranging from near-normal levels (34–37 U/mL) to higher thresholds (100–200 U/mL), and the optimal cutoff remains uncertain. In addition to its role as a categorical risk factor, dynamic changes in CA19-9 during preoperative chemotherapy may provide additional prognostic information. Routine assessment of CA19-9 together with CEA at CRLM diagnosis and during perioperative management may improve risk stratification and guide personalized multidisciplinary treatment strategies.

## Simple Summary

Carbohydrate antigen 19-9 (CA19-9) is a tumor marker commonly used in pancreatic and biliary cancers, but its clinical significance in colorectal liver metastases (CRLM) has not been fully clarified. Increasing evidence suggests that elevated CA19-9 levels are associated with more potentially unfavorable tumor biology and poorer oncologic outcomes in patients undergoing hepatectomy for CRLM. In several studies, CA19-9 has demonstrated stronger prognostic value than carcinoembryonic antigen (CEA) and has been incorporated into modern prognostic models and nomograms. However, the optimal cutoff value of CA19-9 remains controversial, and its interpretation may vary depending on clinical context and treatment stage. In addition, changes in CA19-9 levels during preoperative chemotherapy may provide important information regarding treatment response and postoperative prognosis. This review summarizes current evidence on the prognostic role of CA19-9 in CRLM and discusses its potential utility for improving risk stratification and guiding multidisciplinary treatment strategies in patients with colorectal liver metastases.

## 1. Introduction

Carcinoembryonic antigen (CEA) is a glycoprotein involved in cell adhesion, first described in 1965 in colorectal carcinoma (CRC) tissue and is normally expressed during embryonic development, but re-expressed in malignant conditions, particularly adenocarcinomas of the gastrointestinal tract [1]. Carbohydrate antigen 19-9 (CA19-9) is a sialylated Lewis(a) blood group antigen, first identified in CRC cells but later established as a widely used tumor marker in gastrointestinal malignancies [2].

Several studies have demonstrated the prognostic value of serum CA19-9 in both resectable and unresectable patients with CRC [3,4,5]. In patients with colorectal liver metastases (CRLM), tumor markers play a crucial role in prognostic prediction, evaluation of therapeutic response, and guidance of treatment strategies [1,6,7]. Although almost all earlier studies identified high preoperative CEA levels as strong independent predictors of frequent recurrence and poor survival [8,9,10], most of them did not evaluate CA19-9 levels, limiting their comparative value. In a multicenter study from Japan, the positivity rates of CEA and CA19-9 were 74.2% and 43.8% among all patients with CRLM and 66.3% and 31.6% among those who underwent hepatectomy, respectively, at the time of CRLM diagnosis [11].

We developed and validated nomograms in collaboration with the Japanese Society of Hepato-Biliary-Pancreatic Surgery (JSHBPS) to predict disease-free survival (DFS) after upfront hepatectomy for CRLM [12,13]. Additionally, we reported another nomogram to predict overall survival (OS) after conversion hepatectomy in a French cohort [14]. Interestingly, both nomograms incorporated CA19-9 levels, whereas CEA was not selected as a prognostic variable.

Furthermore, we recently analyzed large-scale data from Japanese patients with CRLM who underwent hepatectomy [11]. Multivariable analysis identified a preoperative CA19-9 level > 100 U/mL as an independent prognostic factor, whereas a CEA level > 100 ng/mL was not. Several studies have demonstrated that, within the same multivariable model, the hazard ratio of CA19-9 for RFS or OS was higher than that of CEA as a prognostic predictor in patients with resectable CRLM [12,13,15,16,17,18,19,20,21,22,23,24].

Taken together, these findings support the concept that CA19-9 is not merely an adjunct tumor marker but a biologically relevant predictor of recurrence and survival in CRLM. In the era of precision oncology, integrating CA19-9 into validated prognostic models may facilitate more accurate recurrence-risk stratification and guide individualized treatment strategies beyond conventional CEA-based assessment.

## 2. Clinical Utility of CA19-9 in the Treatment of Colorectal Cancer

In non-resected CRC cohorts—particularly patients with unresectable metastatic CRC treated with systemic chemotherapy—elevated baseline CA19-9 levels have been correlated with shorter PFS and OS [3]. Moreover, dynamic changes in CA19-9 during chemotherapy have prognostic implications: a marked decline in CA19-9 is associated with improved tumor response and survival, whereas persistently elevated or increasing levels predict poor outcomes [4]. These findings suggest that CA19-9 may function not only as a baseline prognostic biomarker but also as an early indicator of treatment efficacy in mCRC.

In addition, a recent large multicenter study involving 5048 patients with stage II–III CRC who underwent curative resection demonstrated that preoperative CA19-9 levels > 37 U/mL were significantly associated with shorter RFS, particularly among patients with normal CEA levels, and that recurrence risk increased progressively with higher CA19-9 values [5]. Furthermore, a meta-analysis of 17 studies encompassing 6434 CRC patients—treated with surgery alone, surgery plus adjuvant chemotherapy, or systemic chemotherapy—confirmed that elevated pretreatment CA19-9 was significantly associated with poorer OS, pooled PFS, DFS, and RFS [6]. These findings highlight the prognostic significance of CA19-9 in patients with CRC, regardless of resectability status.

## 3. Clinical Utility of CA19-9 in Patients with Colorectal Liver Metastases Undergoing Hepatectomy

Several survival-related endpoints for CRLM are used throughout this manuscript: PFS, defined as the interval from treatment initiation to disease progression or death; DFS, the interval from curative treatment to recurrence or death; RFS, the interval from curative-intent treatment to tumor recurrence (with or without death as an event, depending on the study); and OS, the interval from diagnosis or treatment initiation to death from any cause. The terminology used in this review follows that of the respective original studies.

### 3.1. Prognostic Value of CA19-9 for Recurrence and Long-Term Survival

Serum CA19-9 has been established as an important biomarker reflecting tumor burden and biological aggressiveness in CRLM [6,7]. We searched PubMed for articles published between 1990 and 2025 using the keywords ‘CA19-9,’ ‘CEA,’ ‘CRLM,’ ‘hepatectomy’ (or ‘liver resection’), and ‘prognosis.’ Among the identified studies, we analyzed 12 articles in which both CA19-9 and CEA were simultaneously evaluated (Table 1) [11,15,16,17,18,19,21,22,23,25,26,27].

Eight studies demonstrated a greater prognostic impact of CA19-9 than CEA for DFS, RFS, or OS, whereas only one study reported a significant prognostic role for CEA alone [27]. Two studies showed a significant prognostic impact when the markers were used in combination [22,25]. One study focusing on patients receiving preoperative chemotherapy highlighted the importance of tumor marker normalization after chemotherapy and demonstrated discordant prognostic effects of CA19-9 and CEA for RFS and OS [23]. In several individual studies, CA19-9 was associated with relatively high hazard ratios for RFS or OS compared with other clinical, pathological, and genetic factors. However, in several studies, the timing of tumor marker assessment was not clearly specified, which may limit the interpretation of the results.

In our large-scale Japanese nationwide survey of 2759 patients who underwent initial hepatectomy for CRLM, an elevated CA19-9 level (>100 U/mL) at the time of CRLM diagnosis was the strongest independent predictor of RFS, with hazard ratios of 1.75 [11]. In contrast, CEA was not a significant predictor of either RFS or OS. Moreover, elevated CA19-9 levels identified a subgroup of patients whose oncologic outcomes were comparable to those of borderline-resectable disease, despite having a technically resectable tumor burden [15].

A meta-analysis evaluated the association between preoperative tumor markers and early recurrence (ER) in patients undergoing hepatectomy for CRLM [28]. In this study, ER was most commonly defined as recurrence occurring within 6 months after hepatectomy. In the pooled analysis, both CEA and CA19-9 were significantly associated with an increased relative risk of ER (1.56 for CEA; 1.48 for CA19-9) and the strength of evidence for these associations was graded as moderate (Class II). Importantly, these markers serve as surrogate indicators of tumor burden and potentially unfavorable tumor biology rather than merely reflecting disease volume, thereby complementing morphologic factors such as the number, size, and bilobar distribution of metastases. Although heterogeneity in cut-off values across studies limited the overall level of evidence, the consistent direction of effect suggests that elevated preoperative CEA and CA19-9 levels can identify a subgroup of patients at increased risk of early postoperative relapse.

One unique study evaluated whether “cure” is achievable in patients undergoing hepatectomy for CRLM despite concomitant extrahepatic disease (EHD), defining cure pragmatically as >5-year DFS after the last curative surgery [29]. Among 175 patients with ≥5 years of follow-up (intention-to-treat cohort), 13% (24/175) achieved cure; this increased to 19% (22/109) among those who underwent complete resection of both hepatic and extrahepatic lesions. On multivariable analysis, low CA19-9 (≤37 U/mL) independently predicted cure in both the ITT cohort (RR 8.37) and the EHD-resection cohort (RR 27.4), alongside favorable primary tumor factors (T1–2, and N0 in the EHD-resection cohort), metachronous disease, and a limited total tumor burden (combined liver + EHD tumor count). These findings highlight CA19-9 as a key biologic marker associated with long-term cure even when EHD is present but resectable.

A Japanese multicenter analysis reported that, among patients with BRAF V600E–mutated CRLM undergoing hepatectomy, an elevated preoperative CA19-9 level (≥37 IU/mL) independently predicted significantly worse recurrence-free survival (HR 2.139), supporting the role of CA19-9 as a key biological marker for identifying patients at high risk of very early relapse despite technically curative hepatectomy [30].

Taken together, these findings consistently demonstrate that CA19-9 is a reliable biomarker for predicting recurrence and long-term survival after hepatectomy for CRLM. Routine assessment of CA19-9, together with CEA, is strongly recommended at the time of CRLM diagnosis and at the completion of preoperative chemotherapy.

### 3.2. Scoring Systems and Nomograms Incorporating CA19-9

Scoring systems and nomograms for resected CRLM incorporating CA19-9 were summarized in Table 2 [9,12,14,31,32]. In most models, CA19-9 was retained as an independent predictor and was often assigned a greater weight than conventional clinicopathologic variables. Importantly, several studies demonstrated that CA19-9 remained significant whereas CEA did not, or that composite tumor-marker scores including CA19-9 outperformed CEA alone. The inclusion of CA19-9 improved model discrimination compared with traditional clinical risk scores such as the Fong score [8].

In the JSHBPS multicenter cohort (n = 727) of patients undergoing curative-intent hepatectomy for CRLM, a CA19-9 cutoff ≥ 100 U/mL was a key biomarker of aggressive disease and an independent adverse prognostic factor for both DFS and OS (HR 1.42 and 1.45, respectively) [12]. Importantly, CEA was not a significant factor, and tumor marker levels were assessed at the time of CRLM diagnosis rather than after preoperative chemotherapy. Accordingly, CA19-9 ≥ 100 U/mL was incorporated into the six-variable preoperative nomogram for DFS prediction and assigned 4 points—comparable to extrahepatic disease and a tumor number of 2–4, and greater than a tumor size > 5 cm (2 points). The resulting 0–25-point score enabled clear risk stratification, with the predicted median DFS declining from approximately 8.4 years (0 points) to approximately 1.0 year (10 points) and to <0.6 years (>10 points), supporting the use of CA19-9 to identify patients at high risk of early recurrence.

This JSHBPS nomogram was subsequently validated in 1756 patients undergoing hepatectomy in the modern chemotherapy era and demonstrated moderate discrimination (C-index 0.63) [13]. Furthermore, we proposed the Beppu classification, a three-tier recurrence-risk stratification system for CRLM (low ≤6, moderate 7–10, high ≥11 points), to guide perioperative treatment selection—hepatectomy alone, hepatectomy plus adjuvant chemotherapy, and preoperative chemotherapy followed by hepatectomy, respectively—thereby standardizing decisions regarding the indication and timing of chemotherapy [33]. In a cohort of 173 patients undergoing initial hepatectomy for CRLM, the RAS–Beppu classification (Beppu score plus RAS status) provided clearer recurrence-risk stratification and superior prognostic performance compared with the original Beppu classification and other RAS-incorporating systems, such as the modified Clinical Risk Score (mCRS) and the Genetic and Morphological Evaluation (GAME) score [34].

A French study evaluated 439 patients with initially unresectable CRLM who were treated with conversion chemotherapy followed by hepatectomy and reported that CA19-9 measured immediately before hepatectomy was the most heavily weighted biomarker in a pragmatic nomogram for OS. Using an optimal cut-off of >37 U/mL, elevated CA19-9 remained an independent prognostic factor in multivariable Cox analysis (HR 2.36, *p* < 0.001), outperforming several conventional variables [14]. Accordingly, CA19-9 > 37 U/mL was assigned 10 points—the largest contribution among the five nomogram components—resulting in a total score ranging from 0 to 35. Clinically, the model identified a high-risk group with scores > 16 who had a markedly worse prognosis (5-year OS, 4%) than those with scores ≤ 16 (46.3%), underscoring the central role of CA19-9 as a surrogate marker of potentially unfavorable tumor biology and its value in refining patient selection for hepatectomy after conversion therapy.

One large-scale study uniquely evaluated patients undergoing hepatectomy for CRLM stratified by tumor number (solitary, 2–4, and ≥5) and developed subgroup-specific nomograms for OS [32]. Preoperative CA19-9 ≥ 50 U/mL was an independent adverse prognostic factor in patients with solitary CRLM (HR 2.289, 95% CI 1.593–3.289) and those with 2–4 CRLM (HR 1.863, 95% CI 1.313–2.642), and was incorporated into the respective nomograms with substantial point allocations (55 points for solitary CRLM and 47 points for 2–4 CRLM). In contrast, CA19-9 was not retained as an independent factor in the ≥5 CRLM subgroup. The resulting nomograms demonstrated acceptable discrimination for OS prediction (C-index 0.707 for solitary, 0.695 for 2–4, and 0.687 for ≥5), suggesting that the prognostic impact of CA19-9 is tumor-burden dependent and most informative in patients with a lower metastatic load.

## 4. Optimal Cutoff Values of CA19-9 for Prognostic Prediction in Resected CRLM

The prognostic utility of serum CA19-9 in patients undergoing resection for CRLM has been investigated in several large cohorts; however, the optimal cutoff value remains heterogeneous across studies [9,10,11,12,13,14,15,16,17,18,19,20,21,22,23,24,25,26,27,31,32,33]. Reported preoperative cutoff values can generally be divided into three categories: 34–37 U/mL (near the upper limit of normal), 50–70 U/mL (intermediate cutoff), and 100–200 U/mL (high cutoff). Low to intermediate cutoff values are useful for identifying patients at increased risk of recurrence and poorer prognosis, whereas higher cutoff values may demonstrate a stronger prognostic effect.

Regarding the cutoff values of CA19-9, the reported thresholds remain highly heterogeneous across studies, including upper limits of normal, pre-specified values such as 100 or 200 U/mL, and data-driven values derived from median levels or ROC analyses. Therefore, establishing an optimal cutoff value for CA19-9 remains challenging at present. In studies limited to upfront hepatectomy, the reported cutoff values of CA19-9 at the time of CRLM diagnosis have ranged from 35 to 100 U/mL [12,16,25]. In contrast, although few studies have specifically focused on hepatectomy after preoperative chemotherapy, investigations including conversion cases have demonstrated that normalization of CA19-9 after chemotherapy is an important prognostic factor [14,23]. Further validation studies are warranted to clarify and confirm the clinical significance of these cutoff strategies [13,22,31]. Thus, CA19-9 should be interpreted not only as a categorical risk factor but also as a dynamic biomarker in patients undergoing resection for CRLM.

Tumor burden metrics such as the number of CRLM have been more clearly defined; for both DFS and OS, the three-group classification (1, 2–4, ≥5 lesions) and the two-group classification (1–4 vs. ≥5 lesions) provide the most robust prognostic discrimination, showing very small log-rank *p*-values and the largest hazard ratios among candidate thresholds [35]. However, the most appropriate cutoff for CA19-9 remains uncertain because few studies have specifically aimed to determine its optimal threshold. Further studies are required to establish clinically meaningful CA19-9 cutoff values in this setting.

## 5. Factors Influencing CA19-9 Levels in Patients with CRLM

Serum CA19-9 is a sialylated Lewis(a) blood group antigen widely used as a tumor marker for CRC, including CRLM. Sialylated Lewis(a) is well known to be associated with cancer cell adhesion and metastatic potential [36,37]. Approximately 5–10% of the general population are Lewis antigen-negative and thus unable to synthesize CA19-9 [2]. The prevalence of this phenotype may vary among ethnic groups; however, precise differences remain unclear. In such patients, CA19-9 levels remain undetectable regardless of tumor burden, limiting its utility. For such patients, the clinical utility of DUPAN-II has been established in pancreatic cancer but remains less well defined in CRC [38,39]. Interestingly, Lewis antigen-negative pancreatic cancer has been reported to exhibit a more aggressive phenotype [40], and CA19-9 may still retain some utility as a biomarker in these patients despite the Lewis-negative genotype [41].

Higher CA19-9 levels in CRLM patients often reflect increased tumor burden, particularly in patients with multiple bilobar metastases or concurrent extrahepatic disease [42]. Mucinous histology and tumors with high mucin content tend to produce higher CA19-9 levels [43]. Additionally, KRAS and BRAF mutations have been associated with elevated CA19-9 levels [44].

In contrast, various benign hepatobiliary conditions such as cholestasis, biliary tract inflammation, or liver parenchymal injury can cause transient elevation of CA19-9 level [45,46]. These non-malignant factors may confound CA19-9 interpretation and should be carefully considered in CRLM prognosis assessment. In the perioperative setting, biliary obstruction from tumor compression or postoperative complications such as bile leakage may elevate CA19-9, independent of metastatic progression. To differentiate such false-positive elevations of CA19-9, it may be necessary to relieve biliary obstruction, for example by biliary drainage, followed by repeat measurement of CA19-9 levels.

## 6. Conclusions

CA19-9 has consistently emerged as a robust biomarker reflecting tumor biology in CRLM and provides clinically meaningful prognostic information beyond conventional clinicopathologic factors and CEA, particularly in patients undergoing hepatectomy. Accumulating evidence indicates that elevated preoperative CA19-9 is associated with an increased risk of early recurrence and inferior survival, even among patients with technically resectable disease. Incorporation of CA19-9 into validated prognostic models, such as the JSHBPS nomogram, enables more precise recurrence-risk stratification and supports individualized treatment strategies, including optimal selection and sequencing of surgery and perioperative chemotherapy [12,13,33]. Furthermore, integration with molecular features, as exemplified by the RAS–Beppu classification, enhances prognostic discrimination and facilitates biologically informed clinical decision-making [34].

The current evidence regarding the prognostic role of CA19-9 in resected CRLM is derived predominantly from Japanese cohorts. Compared with Western countries, several factors may influence the generalizability of these findings, including (1) differences in genetic background associated with ethnicity, (2) the higher frequency of routine CA19-9 assessment in Japan, and (3) distinct treatment strategies for CRLM, characterized by more aggressive use of hepatectomy but less frequent use of perioperative chemotherapy and thermal ablation. Therefore, further international validation studies are warranted.

From a clinical standpoint, routine assessment of CA19-9 in combination with CEA at the time of CRLM diagnosis and immediately before hepatectomy should be considered essential for accurate risk stratification and treatment planning. Furthermore, serial measurements of tumor markers are important because they may provide information regarding (1) tumor marker doubling time before upfront hepatectomy [47], (2) tumor response to preoperative chemotherapy, and (3) residual disease or recurrence after hepatectomy. However, CA19-9 values should be interpreted cautiously in the presence of benign hepatobiliary conditions, such as cholestasis or inflammation, which may cause transient elevations.

### Future Perspectives

Future research should focus on the prospective validation of CA19-9-based stratification strategies in the modern multidisciplinary management of CRLM encompassing standard hepatectomy as well as conversion therapy, thermal ablation, and repeat hepatectomy. In particular, the integration of CA19-9 with emerging biomarkers, such as circulating tumor DNA and radiomics, may further improve risk prediction and enable a more comprehensive assessment of tumor biology [48,49,50,51,52].

In addition, the development of dynamic prognostic models incorporating longitudinal changes in CA19-9 and treatment response is warranted. Such approaches may better capture the evolving disease trajectory and refine patient selection for aggressive local therapies. Ultimately, combining serum biomarkers with molecular and imaging data has the potential to advance precision medicine and optimize individualized treatment strategies for patients with CRLM.

## Figures and Tables

**Table 1:** Comparison of CA19-9 and CEA as Prognostic Biomarkers for Recurrence and Long-Term Survival.

No.	Ref.	*n*	Endpoint	Study Population	Timing of Tumor Marker Assessment	CA19-9 Cut-Off	CEA Cut-Off	Hazard Ratio (CA19-9)	Hazard Ratio (CEA)	Other Significant Factors with HRs
1	11	2759	RFS	Upfront, 956; NAC, 1012	At diagnosis of CRLM	100 U/mL	100 ng/mL	1.75	NS	Synchronous CRLM, 1.27; Lymphatic invasion, 1.31; Lymph node metastasis (N1, 1.42; N2/3, 1.44); Number of CRLM ≥ 5, 1.38; non-laparoscopic surgery, 1.24
			OS					1.88	NS	Age ≥ 70, 1.49; Lymph node metastasis (N1, 1.47: N2/3, 1.63); Number of CRLM (2–4), 1.48; Surgical curability (R2), 2.59
2	15	127	OS	Upfront, 48	At diagnosis of CRLM	37 U/mL	Not specified	2.39	NS	None
				NAC, 79				2.24	NS	Maximal diameter > 3 cm, 2.25; right-sided primary tumor, 2.77
3	25	309	OS	Upfront	At diagnosis of CRLM	50 U/mL	25 ng/mL	CA19-9 and/or CEA high, 2.45		No adjuvant chemotherapy, 1.69; Age ≥ 75, 2.09
4	26	205	DFS	Upfront, 89; NAC, 116	Preoperative status (timing relative to NAC: unclear)	39 U/mL	5 U/mL	NS	NS	Resection margin (R1), 1.5; number of CRLM > 2, 1.65; Maximal size of CRLM > 5 cm, 1.67; Primary tumor vascular invasion, 1.65
			OS					2.29	NS	BMI ≥ 24, 1.75; Primary tumor perineural invasion, 1.78
5	16	85	DFS	Upfront	At diagnosis of CRLM	35 U/mL	5 ng/mL	2.91	NS	Synchronous, 2.06; number of CRLM > 1, 2.03; Lymphovascular invasion, 2.06; Ki67 ≥ 70%, 3.19; pMMR, 1.68
			OS					2.28	NS	Lymph node metastasis (N1-2), 2.23; Lymphovascular invasion, 1.79; Ki67 ≥ 70%, 2.70; pMMR, 2.21
6	21	441	RFS	NA	Pre- and post-operative status	37 U/mL	5 ng/mL	Pre, 1.12; Post, 1.09	Pre, NS; Post, 1.17	None
			OS					Pre, 1.13; Post, 1.24	Pre, NS; Post, 1.17	Preoperative YKL-40, 1.19
7	17	580	OS	Upfront, 79; NAC, 355	Pre-hepatectomy	37 U/mL	5 ng/mL	1.47	NS	Age, 1.03; Primary tumor lymph node metastasis, 1.70; Number of CRLM, 1.13; Maximal size of CRLM, 1.07; Extrahepatic metastases, 1.61; R0 resection, 0.56; LDH levels (>ULN), 1.73
8	27	125	OS	NA	Preoperative status (timing relative to NAC: unclear)	34 U/mL	5 ng/mL	NS	1.002	None
9	18	341	OS	Upfront, 178; NAC, 163	Pre-hepatectomy	100 U/mL	20 ng/mL	2.09	NS	Number of CRLM ≥ 4, 1.77; Bilobar CRLM, 1.78; RAS-mutant, 1.73
10	22	787	RFS	NA	Preoperative status (timing relative to NAC: unclear)	200 U/mL	200 ng/mL	Preoperative CEA > 200 ng/mL or CA19-9 > 200 U/mL, 1.33		Primary tumor lymph node metastasis, 1.46; KRAS/NRAS/BRAF mutated, 1.58; mTBS (6–12), 1.49; >12, 2.01); Extrahepatic disease, 1.52
11	19	141	OS	CRLM (≤5) Upfront, 89; NAC, 52	Pre-hepatectomy	100 U/mL	5 ng/mL	1.95	NS	Number of CRLM ≥ 5, 1.91; Bilobar CRLM, 1.78
12	23	62	RFS	POC, 62	Post-POC	37 U/mL	5 ng/mL	2.11	3.08	Radiological non-responder, 2.18
			OS					4.46	NS	Radiological non-responder, 3.14

Abbreviations: Ref., reference; *n*, number of patients; CA19-9, carbohydrate antigen 19-9; CEA, carcinoembryonic antigen; RFS, recurrence-free survival; OS, overall survival; Upfront, upfront hepatectomy; NAC, neoadjuvant chemotherapy; CRLM, colorectal liver metastases; NS, not significant; BR-CRLM, borderline-resectable colorectal liver metastases; BMI, body mass index; Ki-67, Ki-67 labeling index; pMMR, proficient mismatch repair; LDH, lactate dehydrogenase; ULN, upper limit of normal; CRS, clinical risk score; NA, not available; YKL-40, chitinase-3-like protein 1; RAS, rat sarcoma viral oncogene homolog; BRAF, v-Raf murine sarcoma viral oncogene homolog B1; POC, preoperative chemotherapy.

**Table 2:** Prognostic scoring systems and nomograms incorporating CA19-9 for resected CRLM.

No.	Ref.	Model Type and Name	*n*	Study Population	Outcome	Timing of CA19-9 Assessment	CA19-9 Cut-Off	Comparison with CEA	Nomogram Factors or Hazard Ratios (HRs) in Multivariate Analysis	Concordance-Index
1	31	West China Hospital (WCH) nomogram	371 (training) and 161 (validation)	Hepatic resection	OS	Pre-hepatectomy	≥32.3 U/mL	Composite tumor marker superior to CEA alone	HRs KRAS mutant, 1.38; Primary lymph node positive, 1.79; TBS (<3/≥3–9/≥9), 1.36; **TMS (0/1/2)**, 1.52	Training cohort: 0.674; Validation cohort: 0.655
2	32	Nomogram stratified by tumor number	1095	Hepatic resection	OS	Pre-hepatectomy	≥50 U/mL	CEA was a significant factor in only one cohort	HRs Group 1 (Solitary): Right primary site, 1.72; Pulmonary metastases, 1.92; RAS mutation 1.94, **CEA ≥ 200 ng/mL**, 4.44; **CA19-9 ≥ 50 U/m**, 2.29 Group 2 (2–4 tumors): Primary tumor T3–T4, 3.85; Primary lymph node positive, 1.67; Maximal CRLM ≥ 5 cm, 2.00; Pulmonary metastases, 1.71; RAS mutation, 1.46; **CA19-9 ≥ 50 U/m**, 1.86 Group 3 (≥5 tumors): Primary lymph node positive, 1.98; Maximal CRLM ≥ 5 cm, 2.26; RAS mutation, 3.15	0.707 (solitary CRLM); 0.695 (2–4 CRLM); 0.687 (≥5 CRLM)
3	24	Scoring system	295	Initial hepatic resection	Recurrence	Pre-hepatectomy	≥70 U/mL	CEA was not a significant factor	Synchronous, 2; **CA19-9 > 70 U/mL**, 2; Number of CRLM ≥ 5, 1; Maximal CRLM ≥ 5 cm, 2; Resection margin positive; NLR ≥ 2, 2; PNI ≥ 50, 2	ROC AUC: 0.824; 0.721 for CRS; 0.733 for the Beppu nomogram
4	22	Comprehensive Evaluation of Relapse Risk (CERR) Score	787 + 162 (validation)	Curative-intent hepatic resection	RFS	Pre-hepatectomy	>200 U/mL	CEA alone not independently significant	KRAS/NRAS/BRAF-mutated tumor (1 point); Primary lymph node positive (1 point); Extrahepatic disease (1 point); **CEA > 200 ng/mL or CA19-9 > 200 U/mL** (1 point); and mTBS between 5 and 11 (1 point) or 12 and over (2 points)	Training cohort: 0.630; internal validation cohort: 0.736; CRS score: 0.586; GAME score: 0.602
5	14	Imai nomogram	439	Conversion hepatic resection	OS	Post-POC	>37 U/mL	CEA was not a significant factor	Primary lymph node positive, 5; more than six CRLM at hepatectomy, 7; **CA19–9 at hepatectomy >37 U/mL, 10**; disease progression during first-line chemotherapy, 9; and extrahepatic disease, 4	Original cohort: 0.66
6	12	JSHBPS nomogram (Beppu score)	727	Upfront hepatic resection	DFS	At diagnosis of CRLM	>100 U/mL	CEA was not a significant factor	Synchronous, 3; Primary lymph node positive, 3; Number of CRLM (2-4, 4; ≥5, 9); Maximal CRLM > 5 cm; Extrahepatic metastases, 4; **CA19-9 > 100 U/mL**, 4	Original nomogram: 0.66; validation cohort (upfront hepatectomy): 0.63 (13)

Abbreviations: Ref., reference; *n*, number of patients; CA19-9, carbohydrate antigen 19-9; CEA, carcinoembryonic antigen; CRLM, colorectal liver metastases; RAS, rat sarcoma viral oncogene homolog; TBS, tumor burden score; TMS, tumor marker score; AUC, area under the curve; OS, overall survival; CRS, clinical risk score; mCRS, modified clinical risk score; GAME, genetic and morphological evaluation score; NLR, neutrophil-to-lymphocyte ratio; PNI, prognostic nutritional index; RFS, recurrence-free survival; DFS, disease-free survival; BRAF, v-Raf murine sarcoma viral oncogene homolog B1; mTBS, modified tumor burden score; POC, preoperative chemotherapy.

## Data Availability

No new data were created or analyzed in this study. Data sharing is not applicable to this article.
